# Pediatric Multiorgan Lung Transplantation: Four Decades of National Trends and Outcomes

**DOI:** 10.1111/petr.70466

**Published:** 2026-10-15

**Authors:** Carl Britto, Ali Demir, Eleanor D. Muise, J. Brian McAlvin, Don Hayes, Levent Midyat

**Affiliations:** ^1^ Division of Medical Critical Care, Boston Children's Hospital, Department of Pediatrics, and Margaret C Ryan Global Health Program Boston Children's Hospital Boston Massachusetts USA; ^2^ Division of Pulmonary Medicine University of Pittsburgh School of Medicine, Children's Hospital of Pittsburgh Pittsburgh Pennsylvania USA; ^3^ Division of Pediatric Pulmonary Medicine Hassenfeld Children's Hospital, NYU Langone Health New York New York USA; ^4^ Division of Medical Critical Care Boston Children's Hospital Boston Massachusetts USA; ^5^ The Heart Institute, Cincinnati Children's Hospital Medical Center Cincinnati Ohio USA; ^6^ Division of Pulmonary Medicine Cincinnati Children's Hospital Medical Center Cincinnati Ohio USA; ^7^ Department of Pediatrics University of Cincinnati College of Medicine Cincinnati Ohio USA

**Keywords:** graft survival, longitudinal trends, lung transplantation, multiorgan transplantation, national registry

## Abstract

**Background:**

Pediatric lung transplantation (LTx) and multiorgan lung transplantation (MOLT) remain uncommon, and national data describing temporal trends and outcomes are limited. We characterized four decades of MOLT in the United States, with particular emphasis on the evolving pediatric experience.

**Methods:**

We analyzed Organ Procurement and Transplantation Network Standard Transplant Analysis and Research files provided by the United Network for Organ Sharing from 1987 to 2024. The cohort included 54 797 isolated LTx recipients and 1 963 MOLT recipients, classified into mutually exclusive groups of dual‐organ lung‐heart (*n* = 1557), lung‐liver (*n* = 216), lung‐kidney (*n* = 154), and other multiorgan configurations (*n* = 36). Cox regression, Fine–Gray competing‐risks, landmark, and interaction analyses evaluated graft‐failure associations across four transplant eras and seven age groups.

**Results:**

Pediatric transplant volume declined substantially, comprising 0.8% of isolated LTx procedures in 2020–2024. Lung‐heart transplantation decreased from 9.0% of lung‐involved procedures before 2000 to 1.8% in 2020–2024, whereas lung‐liver and lung‐kidney transplantation increased modestly, predominantly among adults. Pretransplant ECMO use increased across cohorts, reaching 27.6% among lung‐heart and 27.8% among lung‐kidney recipients in 2020–2024. One‐year graft survival improved progressively across eras.

**Conclusions:**

Pediatric MOLT has evolved within a changing national lung‐transplant landscape characterized by declining pediatric volume, shifting procedural patterns, increasing recipient acuity, and improving early graft outcomes. Continued evaluation is needed as long‐term follow‐up from recent eras matures.

## Graphical abstract

This national retrospective registry‐based cohort study included 56 760 lung transplant recipients in the United States from 1987 to 2024. Pediatric transplant volume and lung‐heart transplantation declined, whereas lung‐liver and lung‐kidney transplantation increased predominantly among adults. Despite increasing recipient acuity, one‐year graft survival improved across transplant eras.

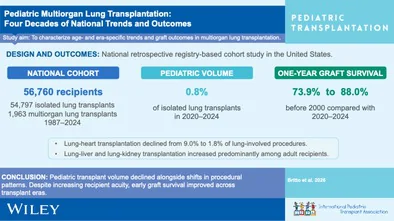

**Abbreviations**
BOS: Bronchiolitis Obliterans Syndrome
CF: Cystic Fibrosis
CFTR: Cystic Fibrosis Transmembrane Conductance Regulator
CI: Confidence Interval
CLAD: Chronic Lung Allograft Dysfunction
COVID‐19: Coronavirus Disease 2019
cPRA: Calculated Panel Reactive Antibody
DCD: Donation After Circulatory Death
ECMO: Extracorporeal Membrane Oxygenation
ETI: Elexacaftor/Tezacaftor/Ivacaftor
FDA: U.S. Food and Drug Administration
HR: Hazard Ratio
IQR: Interquartile Range
LTx: Lung Transplantation
MOLT: Multiorgan Lung Transplantation
OPTN: Organ Procurement and Transplantation Network
PGD: Primary Graft Dysfunction
PTLD: Post‐Transplant Lymphoproliferative Disorder
RAS: Restrictive Allograft Syndrome
SD: Standard Deviation
sHR: Subdistribution Hazard Ratio
STAR: Standard Transplant Analysis and Research Registry
UNOS: United Network for Organ Sharing

## 1 Introduction

The field of lung transplantation (LTx) in the United States has evolved due to shifting recipient demographics, changes in allocation policies, and advances in surgical and intensive care practices, including disease‐specific and post‐transplant management. A small but clinically important subset of candidates undergo combined lung transplantation with another solid organ, reflecting the multisystem burden of advanced lung disease [1, 2, 3, 4, 5]. Although multiorgan lung transplantation (MOLT) remains uncommon, its indications and recipient characteristics have evolved substantially over time. Recent changes in pediatric lung transplantation have been particularly striking, including a marked decline in transplant volume and a fundamental shift in diagnostic indications, with pulmonary hypertension surpassing cystic fibrosis (CF) as the leading indication for the first time in 2020, coinciding with the introduction of highly effective CFTR modulator therapies [2, 6]. Whether these changes have similarly influenced pediatric MOLT remains unclear.

As transplant candidacy expands to increasingly complex patients requiring combined organ transplantation, understanding these changes is important for characterizing the evolving landscape of MOLT and its outcomes. Pediatric multiorgan transplantation presents unique challenges, including differences in underlying disease etiology, surgical complexity, immunologic responses, and developmental considerations; however, national data describing long‐term trends and outcomes remain limited [7, 8].

Using national United Network for Organ Sharing (UNOS) data, we aimed to characterize four decades of national trends in MOLT, with a particular focus on the evolving pediatric experience while comparing recipient characteristics and post‐transplant outcomes across age groups [4, 8, 9].

## 2 Methods

### 2.1 Study Design and Data Source

This study was deemed exempt by the local Institutional Review Board. De‐identified data were obtained from the Organ Procurement and Transplantation Network (OPTN) Standard Transplant Analysis and Research (STAR) files provided by the United Network for Organ Sharing (UNOS).

In this national retrospective cohort study, we included LTx recipients in the United States between January 1, 1987, and December 31, 2024. A total of 54 797 isolated lung transplant recipients and 1 963 MOLT recipients were included, comprising mutually exclusive groups of dual‐organ lung‐heart (*n* = 1557), lung‐liver (*n* = 216), lung‐kidney (*n* = 154), and other multiorgan configurations (*n* = 36) (Figure S1). For organ‐involved descriptive analyses, triple‐organ recipients were included in each applicable organ‐specific group; therefore, these groups were not mutually exclusive. Transplants were stratified across four transplant eras (1987–1999, 2000–2009, 2010–2019, and 2020–2024) and seven developmental/demographic age groups (< 1, 1–5, 6–10, 11–17, 18–35, 36–59, and ≥ 60 years), with binary pediatric (< 18 years) versus adult (≥ 18 years) categorization used for select analyses. Patient selection criteria are described in the Supporting Information.

### 2.2 Variables and Outcomes

Indicators for disease severity prior to transplant included the presence of mechanical ventilation and extracorporeal membrane oxygenation (ECMO), which were analyzed as categorical variables. Total ischemic time was calculated as the interval between donor cross‐clamp and recipient reperfusion and analyzed as a continuous variable (hours). Calculated panel reactive antibody (cPRA) values were analyzed as continuous variables, with availability limited to more recent transplant eras. Post‐transplant malignancy, new‐onset diabetes mellitus, and chronic dialysis requirement were defined by the presence of any corresponding UNOS‐reported complication during follow‐up and analyzed as time‐fixed covariates indicating occurrence at any point during available follow‐up. Because the timing of these complications was not modeled, their associations with graft failure were considered exploratory and were not interpreted as causal.

The primary outcome was time to lung graft failure. Reported causes of graft failure, including primary graft dysfunction (PGD), acute rejection, and chronic lung allograft dysfunction (CLAD; encompassing bronchiolitis obliterans syndrome [BOS] and restrictive allograft syndrome [RAS]), were identified using UNOS cause‐of‐failure codes. For primary survival analyses, graft failure was defined as the earliest occurrence of documented graft failure, retransplantation, or death from any cause. Failure of accompanying non‐pulmonary allografts was not analyzed separately. Cause of death was extracted from primary, secondary, and tertiary UNOS fields, treated equivalently. Individual decedents could therefore contribute more than one cause‐of‐death record, and cause categories were not mutually exclusive. For competing‐risk analyses, documented graft failure or retransplantation was the event of interest, death without prior graft failure or retransplantation was treated as the competing event, and recipients alive with a functioning graft were censored.

### 2.3 Statistical Analysis

Continuous variables were reported as mean ± SD or median (IQR), and categorical variables as counts and percentages. Graft survival was estimated using Kaplan–Meier methods, with differences assessed by log‐rank testing. Multivariable Cox proportional hazards regression was used to evaluate associations with graft failure among isolated lung transplant recipients; covariates included transplant era, age group, primary diagnosis, pretransplant mechanical ventilation, pretransplant ECMO, and ischemic time, and model discrimination was assessed using Harrell's C‐index. Separate unadjusted and adjusted Cox models compared graft failure across transplant types, with isolated lung transplantation as the reference group; adjusted models included age group, transplant era, primary diagnosis, pretransplant mechanical ventilation, and pretransplant ECMO. Additional exploratory Cox models evaluated associations between post‐transplant malignancy, diabetes, and dialysis and graft failure. The proportional hazards assumption was evaluated using Schoenfeld residuals and formal testing; because violations were observed, hazard ratios were interpreted cautiously. Age‐stratified models estimated associations within each developmental/demographic age group. Given the limited number of events in several rare MOLT subgroups, these analyses were considered exploratory. Competing risks were addressed using Fine–Gray subdistribution hazard regression, with baseline covariates only; post‐transplant complications were excluded. A landmark analysis restricted to 1‐year survivors evaluated conditional graft survival. Interactions between transplant type and age group and between transplant type and transplant era were evaluated using likelihood ratio tests. Complete‐case analyses were performed for multivariable models. All analyses were performed in R version 4.5.2 using the *survival* and *tidycmprsk* packages. Missingness was low for most variables, with details regarding data completeness and missing‐data handling provided in the Supporting Information.

## 3 Results

### 3.1 Cohort Characteristics and Procedural Distribution

A total of 56 760 recipients underwent LTx, including 54 797 isolated LTx recipients and 1 963 MOLT recipients, comprising mutually exclusive groups of dual‐organ lung‐heart (*n* = 1557), lung‐liver (*n* = 216), lung‐kidney (*n* = 154), and other multiorgan configurations (*n* = 36). Among isolated lung recipients, transplant volume increased progressively across eras, driven primarily by adult recipients (Table 1, Figure 1). In contrast, pediatric transplant volume declined substantially following the introduction of highly effective CFTR modulator therapy in the United States in late 2019.

**TABLE 1:** Characteristics of isolated lung and multiorgan lung transplants in adult and pediatric recipients.

Parameter	Era	Isolated organ	Lung‐heart	Lung‐liver	Lung‐kidney
Total *N* ^a^	1987–1999	7036	700	10	4
	2000–2009	14 455	372	44	14
	2010–2019	21 872	300	104	76
	2020–2024	11 434	214	76	79
Re‐transplantation (%)	1987–1999	232 (3.3%)	23 (3.3%)	0 (0%)	0 (0%)
	2000–2009	637 (4.4%)	7 (1.9%)	1 (2.3%)	6 (42.9%)
	2010–2019	858 (3.9%)	7 (2.3%)	0 (0%)	24 (31.6%)
	2020–2024	394 (3.4%)	6 (2.8%)	1 (1.3%)	12 (15.2%)
Waitlist time, days (median [IQR])	1987–1999	234 (91–461)	205 (71–474)	119 (50–252)	428 (304–565)
	2000–2009	138 (37–429)	146 (52–414)	164 (70–350)	86 (39–175)
	2010–2019	52 (15–161)	70 (19–206)	114 (36–296)	28 (10–77)
	2020–2024	30 (10–87)	36 (11–123)	46 (16–126)	35 (11–74)
Functionally disabled at TX (%)	1987–1999	2944 (41.8%)	445 (63.6%)	4 (40%)	0 (0%)
	2000–2009	1551 (10.7%)	101 (27.2%)	13 (29.5%)	2 (14.3%)
	2010–2019	644 (2.9%)	35 (11.7%)	8 (7.7%)	0 (0%)
	2020–2024	427 (3.7%)	11 (5.1%)	1 (1.3%)	1 (1.3%)
Ventilation before TX (%)	1987–1999	282 (4%)	40 (5.7%)	1 (10%)	0 (0%)
	2000–2009	759 (5.3%)	34 (9.1%)	5 (11.4%)	1 (7.1%)
	2010–2019	1410 (6.4%)	33 (11%)	11 (10.6%)	18 (23.7%)
	2020–2024	627 (5.5%)	21 (9.8%)	5 (6.6%)	8 (10.1%)
ECMO before TX (%)	1987–1999	24 (0.3%)	7 (1%)	0 (0%)	0 (0%)
	2000–2009	130 (0.9%)	21 (5.6%)	0 (0%)	0 (0%)
	2010–2019	1159 (5.3%)	65 (21.7%)	2 (1.9%)	16 (21.1%)
	2020–2024	910 (8%)	59 (27.6%)	3 (3.9%)	22 (27.8%)
Distancetraveled, miles (median [IQR])	1987–1999	87 (8–271)	68 (6–188)	37 (2–62)	62 (25–142)
	2000–2009	114 (17–303)	72 (14–221)	48 (2–118)	2 (0–15)
	2010–2019	152 (29–316)	126 (22–306)	74 (11–177)	102 (24–187)
	2020–2024	248 (101–571)	266 (98–380)	264 (97–573)	201 (64–651)
Ischemic time, hours (median [IQR])	1987–1999	4.2 (3.2–5.5)	3.4 (2.6–4.2)	4.5 (3.2–5.2)	4.8 (4.8–4.9)
	2000–2009	4.8 (3.8–5.9)	3.9 (3.2–4.6)	5.1 (3.2–6.2)	4.8 (3.9–5.3)
	2010–2019	5.2 (4.2–6.3)	3.8 (3.1–4.4)	4.8 (3.9–5.8)	4.9 (4–5.9)
	2020–2024	6.3 (5.1–8.2)	4.1 (3.5–4.8)	5.8 (4.4–7.1)	5.8 (4.8–7)
Length of stay, days (median [IQR])	1987–1999	16 (11–27)	53 (32–64)	94 (94–94)	NA (NA‐NA)
	2000–2009	15 (10–26)	23 (13–48)	28 (13–43)	34 (20–72)
	2010–2019	17 (12–29)	29 (17–54)	32 (21–60)	31 (15–58)
	2020–2024	20 (14–34)	32 (19–51)	28 (18–45)	37 (21–55)
Graft failure (%)	1987–1999	6492 (92.3%)	630 (90%)	8 (80%)	4 (100%)
	2000–2009	12 600 (87.2%)	285 (76.6%)	30 (68.2%)	12 (85.7%)
	2010–2019	12 809 (58.6%)	150 (50%)	47 (45.2%)	36 (47.4%)
	2020–2024	1881 (16.5%)	53 (24.8%)	14 (18.4%)	19 (24.1%)
Post‐TX Diabetes (%)	1987–1999	1861 (26.4%)	4 (0.6%)	0 (0%)	0 (0%)
	2000–2009	5023 (34.7%)	22 (5.9%)	12 (27.3%)	4 (28.6%)
	2010–2019	3075 (14.1%)	51 (17%)	47 (45.2%)	36 (47.4%)
	2020–2024	590 (5.2%)	39 (18.2%)	27 (35.5%)	22 (27.8%)

^a^ Lung‐heart, lung‐liver, and lung‐kidney columns represent organ‐involved procedures and are not mutually exclusive; higher‐order multiorgan recipients may be included in more than one column.

**FIGURE 1:**
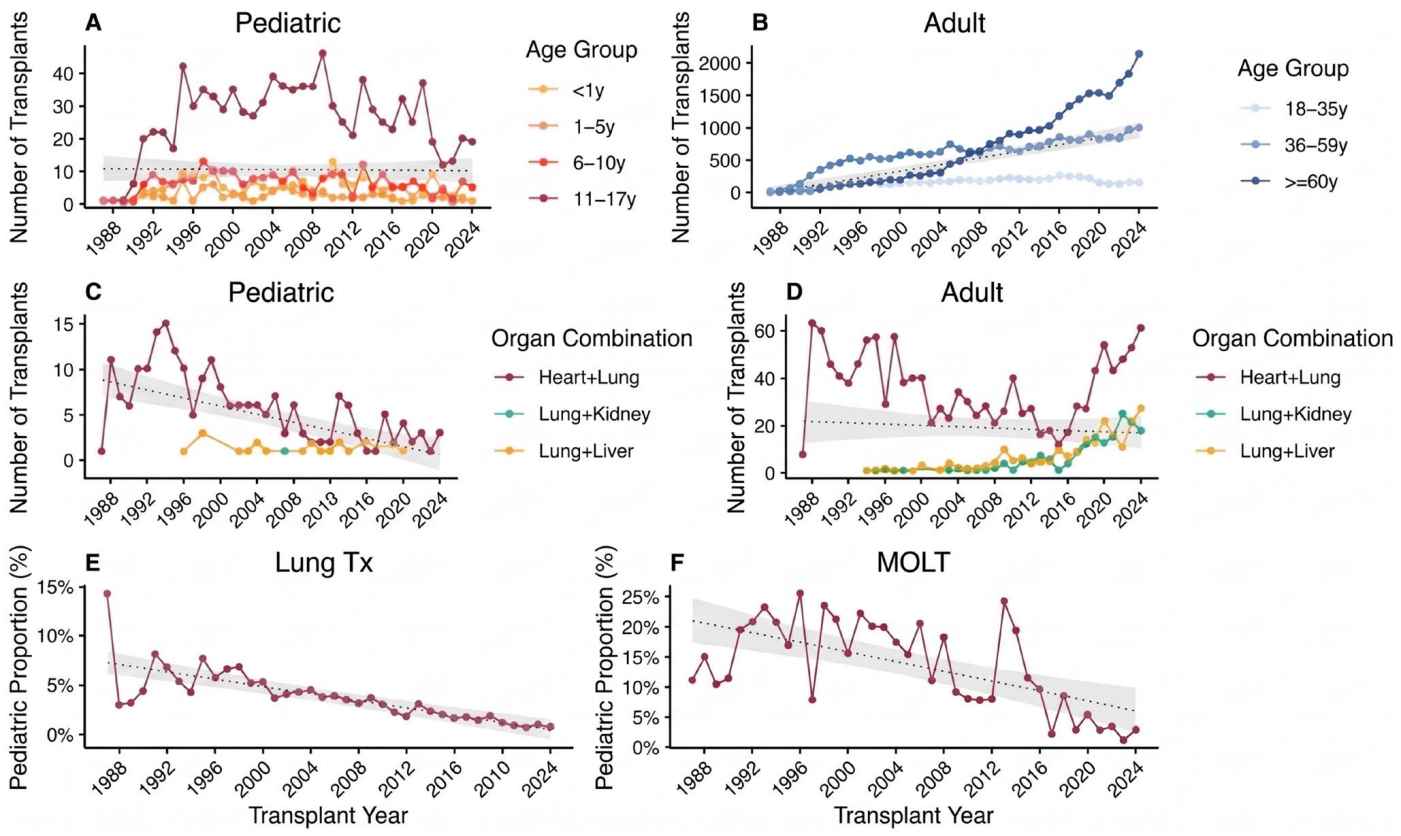
Longitudinal trends in isolated and multiorgan lung transplant volume among pediatric and adult recipients, 1987–2024. Panels A and B: Annual transplant volume for pediatric (A) and adult (B) isolated lung transplant recipients, stratified by age group. Pediatric age groups are represented by warm tones and adult age groups by cool tones. Panels C and D: Annual transplant volume for lung‐heart, lung‐liver, and lung‐kidney organ‐involved procedures among pediatric (C) and adult (D) recipients. For organ‐involved descriptive analyses, recipients in the other multiorgan configurations group were included in each applicable organ‐specific group; therefore, these groups were not mutually exclusive. Panels E and F: Annual percentage of total isolated lung (E) and multiorgan lung (F) transplant volume comprising pediatric recipients aged < 18 years. In Panels A–D, individual points represent absolute annual counts; in Panels E and F, points represent annual percentages. Black dashed lines represent cohort‐wide trends estimated using linear regression, with 95% confidence intervals shown by gray ribbons.

Lung‐heart transplantation comprised the majority of MOLT procedures (Figure 1C,D). Pediatric recipients represented a substantial proportion of early lung‐heart transplantation. Lung‐heart transplantation was most frequent in the pre‐2000 era among middle‐aged (*n* = 312) and young adult (*n* = 267) recipients. A total of 234 lung‐liver‐involved transplants were identified in the OPTN STAR database, including 216 dual‐organ lung‐liver transplants and 18 other multiorgan configurations involving the liver. Lung‐liver transplantation remained rare among pediatric recipients, with 3 cases before 2000, 5 in 2000–2009, 8 in 2010–2019, and 1 in 2020–2024. In contrast, among recipients aged 36–59 years, lung‐liver transplantation increased across eras, from 3 procedures before 2000 to 9 in 2000–2009, 30 in 2010–2019, and 44 in 2020–2024. Among recipients aged ≥ 60 years, lung‐liver transplantation expanded markedly in the modern era, increasing from 1 procedure in 2000–2009 and 9 in 2010–2019 to 42 in 2020–2024.

Other multiorgan transplant configurations were rare. Lung‐kidney‐heart (*n* = 16) and lung‐liver‐heart (*n* = 14) were the most common, while lung‐liver‐kidney (*n* = 4), lung‐liver‐pancreas (*n* = 2), and lung‐pancreas (*n* = 3) occurred sporadically without consistent temporal patterns. For organ‐involved descriptive analyses, recipients in the other multiorgan configurations group were included in each applicable organ‐specific group; therefore, these groups were not mutually exclusive.

### 3.2 Longitudinal Volume Trends and Demographic Shift

Single‐organ transplantation predominated across all eras and age groups (Figure 1, Table 1); however, the relative contribution of multiorgan procedures varied by both age and transplant era. Historically, lung‐heart transplantation comprised the largest share of multiorgan procedures (Figure 1C), representing 23.5%–46.9% of all lung‐involved transplants among pediatric recipients aged ≤ 10 years in the pre‐2000 era. This proportion declined substantially in the 2020–2024 era, falling to 9.1%–13.0% within the same age groups (Figure S2).

A similar pattern was observed in adults (Figure 1D; Figure S2). Among recipients aged 18–35 years, lung‐heart transplantation decreased from 17.1% of lung‐involved procedures pre‐2000 to 7.9% post‐2020, while in those aged 36–59 years, it declined from 6.8% to 3.1%. In contrast, lung‐liver and lung‐kidney transplantation remained uncommon throughout the study period but increased modestly in the adult cohorts. Lung‐kidney transplants increased from 0.1% pre‐2000 to 1.9% in 18–35‐year recipients and 1.1% in 36–59‐year recipients post‐2020. Among recipients ≥ 60 years, multiorgan transplantation remained rare overall but became an established component of practice in the modern era, with lung‐heart accounting for 0.5%, lung‐kidney 0.4%, and lung‐liver 0.5% of lung‐involved procedures post‐2020 (Figure S3).

### 3.3 Diagnostic Indications Across the Lifespan

Indications for transplantation shifted predictably with age (Figure 2). Among isolated lung transplant recipients, infants were primarily transplanted for pulmonary hypertension (*n* = 29) and pulmonary vascular disease (*n* = 20). CF predominated among pediatric and young adult recipients (6–10 years: *n* = 96; 11–17 years: *n* = 554; 18–35 years: *n* = 3438). Chronic obstructive pulmonary disease was the primary indication in middle‐aged adults (36–59 years; *n* = 6001), whereas idiopathic pulmonary fibrosis predominated among recipients aged ≥ 60 years (*n* = 10 757).

**FIGURE 2:**
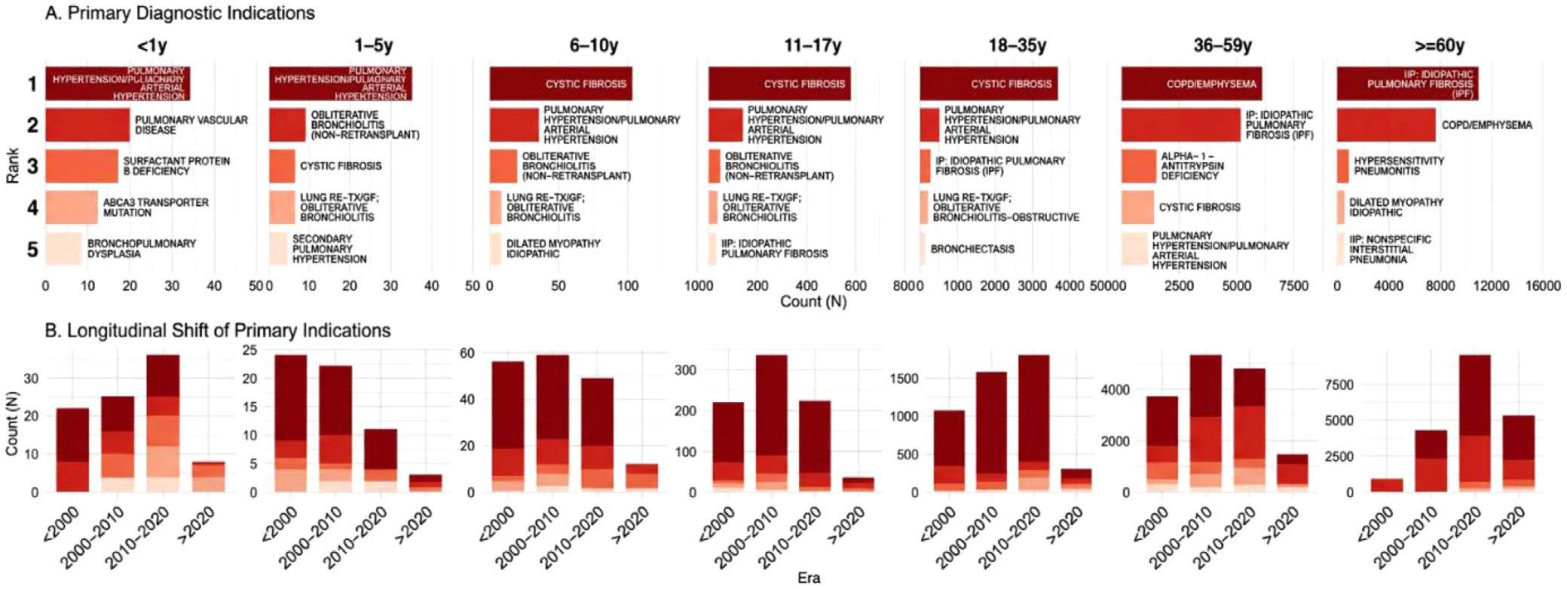
Dominant clinical indications and longitudinal diagnostic transitions across the lifespan. Panel A: Primary diagnostic indications. Horizontal bar charts display the five most common primary indications for lung transplantation across seven age groups. Rankings are based on absolute transplant volume within each age stratum. Panel B: Longitudinal shift in primary indications. Stacked bar charts display the distribution of the five most common primary indications across four transplant eras: 1987–1999, 2000–2009, 2010–2019, and 2020–2024. Values represent absolute transplant counts within each age‐by‐era stratum.

In lung‐heart recipients, pulmonary arterial hypertension was the leading indication in both adult (*n* = 325) and pediatric recipients (*n* = 68), whereas congenital heart disease represented a major indication in the pediatric population (*n* = 22). For lung‐liver transplantation, CF was the leading indication in both adult (*n* = 75) and pediatric recipients (*n* = 16), together with idiopathic pulmonary fibrosis in adults (*n* = 52). In lung‐kidney recipients, CF was the leading indication in adults (*n* = 20), followed by pulmonary fibrosis from other specified causes (*n* = 19). Pediatric lung‐kidney transplantation was exceedingly rare, with only one case identified in the cohort.

CF demonstrated the most striking temporal change in transplant indications. Annual CF transplant volume peaked between 2010 and 2018, reaching 122–179 transplants annually among recipients aged 18–35 years and 50–77 among those aged 36–59 years. Following the widespread availability of elexacaftor/tezacaftor/ivacaftor (ETI) in the United States in 2019, CF lung transplant volume declined by more than 80%, falling to 22–32 annually in recipients aged 18–35 years and 12–15 in those aged 36–59 years by 2022–2024. Pediatric CF lung transplantation became exceptionally uncommon after 2021, with only 1–3 cases performed annually across all pediatric age groups.

### 3.4 Pre‐Transplant Recipient Characteristics

Across isolated and multiorgan procedures, pediatric LTx recipients (*n* = 1594) had substantially greater pre‐transplant acuity than adults across multiple domains (Table 1; Table S1). Functional disability was nearly universal among pediatric recipients in recent eras, whereas adult recipients demonstrated markedly lower rates overall, with higher disability observed among lung‐heart recipients than isolated lung recipients.

Mechanical ventilation was consistently highest among infants across eras and remained uncommon in adult LTx recipients. Ventilator dependence increased modestly over time among adult age groups. Across transplant types, MOLT recipients generally showed higher ventilator dependence than isolated LTx recipients.

ECMO support as a bridge to transplantation increased dramatically over time across all age groups. Among young adults, ECMO at the time of transplant increased from 0.5% (*n* = 7) pre‐2000 to 26.6% (*n* = 150) post‐2020. ECMO in middle‐aged adults increased from 0.2% (*n* = 8) to 13.6% (*n* = 494), and adolescents rose from 0.4% to 23.4%. Pediatric ECMO utilization reached 20%–50% in certain subgroups in recent eras. ECMO was highest in lung‐kidney recipients aged 18–35 years (37.1%) and lung‐heart recipients ≥ 60 years (20.7%), compared with substantially lower rates in isolated lung recipients. In adolescents, ECMO was required in 12.5% (*n* = 15) of lung‐heart recipients versus 5.4% (*n* = 53) of LTx recipients.

Waitlist times shortened dramatically in adolescents and adults (228 and 258 days pre‐2000 to 36 and 32 days post‐2020) but increased modestly in infants (20 to 82 days) and 1–5‐year‐olds (58 to 107 days). Among multiorgan recipients, lung‐liver recipients aged 18–35 years experienced the longest waitlist times (244 days [IQR 73–469]), while lung‐kidney recipients in the same age group had the shortest (41 days [IQR 12–68]) (Table 1).

Ischemic times increased across transplant eras in both adult and pediatric recipients. In adults, ischemic duration increased from approximately 3.8–5.2 h pre‐2000 to 6.2–6.8 h post‐2020, while pediatric recipients demonstrated a rise from 5.0–5.1 to 5.9–6.3 h. Within adult subgroups, median ischemic time increased from 4.0 h (IQR 3.1–5.2) to 6.5 h (IQR 5.3–8.4) in middle‐aged recipients, from 3.8 h (IQR 3.0–4.8) to 6.2 h (IQR 4.9–8.1) in recipients aged ≥ 60 years, and from 5.2 h (IQR 3.9–6.5) to 6.8 h (IQR 5.4–8.5) in young adults. Median ischemic times were shorter in lung‐heart procedures compared with isolated LTx.

Allosensitization varied by age and transplant type. In the 2020–2024 era, mean cPRA ranged from 11.6 ± 23.3 in recipients aged ≥ 60 years to 34.2 ± 40.3 in children aged 1–5 years. Among MOLT recipients, mean cPRA was highest in lung‐kidney recipients aged 18–35 years (19.7 ± 34.7). Pre‐transplant creatinine was markedly elevated in lung‐kidney recipients (3.16–4.35 mg/dL) compared with other cohorts.

### 3.5 Early Post‐Transplant Outcomes

Length of stay was consistently longer in multiorgan recipients compared with isolated lung recipients. Infants experienced the longest hospitalizations overall, with median stays of 70 days (IQR 52–103) after lung‐heart transplantation and 51 days (IQR 33–80) after isolated LTx. Among recipients aged 36–59 years, lung‐heart recipients experienced a median hospital stay of 28 days (IQR 18–45), compared with 18 days (IQR 12–30) for isolated LTx.

Observed graft‐failure proportions decreased across organ combinations and age groups over time, although comparisons involving the 2020–2024 era were limited by shorter available follow‐up. In the most recent era, multiorgan graft‐failure rates marginally exceeded those of isolated LTx, except for lung‐liver transplantation, which was comparable (18.4% versus 16.5%). Among pediatric recipients, higher graft‐failure rates observed after MOLT in earlier eras largely converged with isolated LTx in the modern era. Among older adults, graft‐failure rates remained higher after MOLT than isolated LTx. Primary graft dysfunction remained uncommon across transplant combinations.

Acute rejection was common in early eras but became relatively rare after 2010. Chronic rejection deaths peaked in 2000–2009. Death attributable to chronic rejection was less frequent in all MOLT cohorts (2.4%–8.3%) than in isolated LTx (7.5%–22.2%). Retransplantation remained relatively stable in isolated lung and lung‐heart recipients but was markedly higher among lung‐kidney recipients, particularly in earlier eras.

Observed rates of dialysis, diabetes, and malignancy were lower in the 2020–2024 era, although these comparisons were limited by shorter available follow‐up. Post‐transplant diabetes declined from a 2000–2009 peak of 45.6% in adolescents and 34.0% in recipients aged 36–59 years to 4.8%–12.5% across adolescent and adult groups in 2020–2024, while malignancy declined from 35.1% among recipients aged ≥ 60 years in 2000–2009 to 5.3% in 2020–2024. Post‐transplant diabetes was more frequent among lung‐liver (35.5%) and lung‐kidney (27.8%) recipients in the modern era than among isolated lung (5.2%) and lung‐heart (18.2%) recipients. Post‐transplant dialysis remained uncommon after isolated LTx but was more frequent among lung‐heart recipients aged ≥ 60 years (13.4%, *n* = 11) and lung‐kidney recipients aged 18–35 years (25.7%, *n* = 9). Among recipients aged ≥ 60 years, post‐transplant malignancy increased through 2000–2009 before declining substantially in the most recent era.

Neurodevelopmental outcomes showed marked age‐specific vulnerability. Cognitive delay occurred in 21.1% (*n* = 278) of infants and 6.9% (*n* = 38) of children aged 1–5 years after isolated LTx, compared with 10.6% (*n* = 11) and 5.2% (*n* = 10), respectively, among lung‐heart recipients. In contrast, 82% of young adults returned to normal functional status.

### 3.6 Survival Analysis

Graft survival improved substantially across transplant eras. Among 53 962 recipients included in the survival analysis, median follow‐up was 3.4 years (range 0–34.8), and 33 556 recipients (62.2%) experienced graft failure. One‐year graft survival increased from 73.9% before 2000 to 82.3%, 87.9%, and 88.0% across successive eras. Longer‐term comparisons involving the 2020–2024 era were limited by shorter available follow‐up.

In multivariable analyses, more recent transplant eras were independently associated with lower graft failure risk. Pulmonary vascular disease and re‐transplantation were associated with increased risk, whereas ischemic time and recipient sex were not independently associated. Mechanical ventilation and ECMO were associated with higher graft failure risk. In exploratory analyses, post‐transplant malignancy, chronic dialysis, and diabetes were also associated with graft failure; these associations were not interpreted as causal. In analyses including all transplant types, transplant configuration was also independently associated with graft failure. Compared with isolated lung transplantation, lung‐liver transplantation was associated with lower graft failure risk in both unadjusted (HR 0.74, *p* = 0.003) and adjusted analyses (HR 0.80, *p* = 0.033), while lung‐heart showed borderline benefit after adjustment (HR 0.94, *p* = 0.052) and lung‐kidney showed no significant association (Figure 3).

**FIGURE 3:**
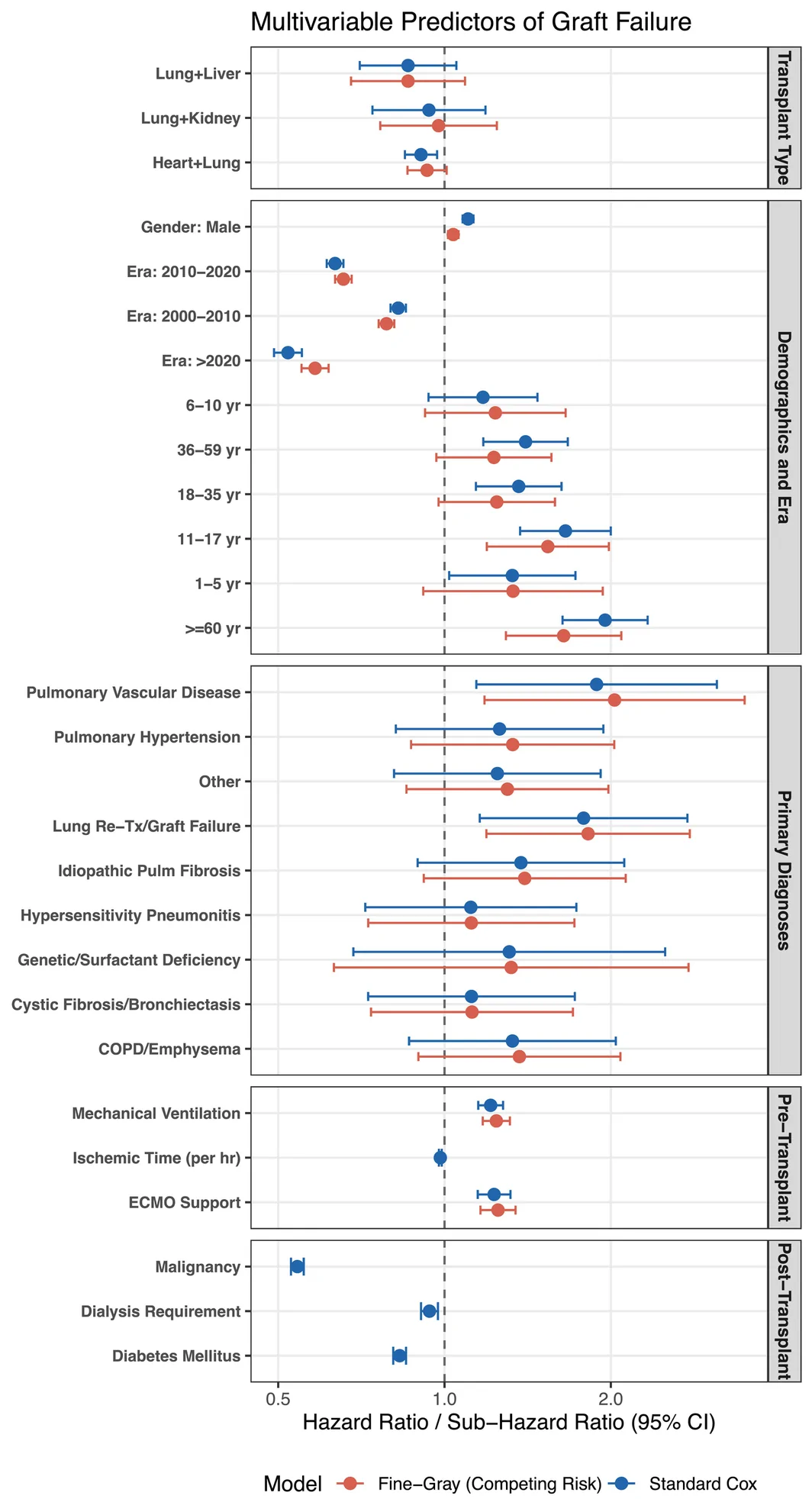
Risk profile for lung graft failure. Forest plot comparing adjusted hazard ratios (HRs; Cox model, blue) and subdistribution hazard ratios (sHRs; Fine–Gray model, red) for graft failure. Variables are grouped by transplant era, demographic characteristics, pretransplant factors, and post‐transplant complications. The dashed line indicates no association (HR or sHR = 1.0). Graft‐failure risk decreased progressively across transplant eras, with the largest reduction in 2020–2024 (sHR 0.58; 95% CI 0.55–0.62; *p* < 0.001). Adolescents and recipients aged ≥ 60 years had higher risk, with attenuation in the Fine–Gray model after accounting for competing mortality. Bronchopulmonary dysplasia, genetic or surfactant deficiencies, pulmonary hypertension, retransplantation, mechanical ventilation, and ECMO were associated with higher risk. Post‐transplant malignancy, diabetes, and dialysis were evaluated as exploratory time‐fixed covariates in the Cox model and were excluded from the Fine–Gray model.

Multiorgan effects varied by era. Compared with isolated lung transplantation, lung‐heart transplantation was not associated with graft failure in the pre‐2000 (HR 1.03, *p* = 0.467) or 2010–2019 (HR 0.99, *p* = 0.887) eras, was associated with lower risk in 2000–2009 (HR 0.86, *p* = 0.020), and higher risk in the post‐2020 era (HR 1.52, *p* = 0.004). Age‐specific associations were also observed, with lower adjusted graft failure risk among infants (HR 0.62, *p* = 0.042) and children aged 6–10 years (HR 0.65, *p* = 0.047), but not among adults. Lung‐liver transplantation was associated with lower graft failure risk in adolescents (HR 0.45, *p* = 0.034) and higher risk in children aged 6–10 years (HR 3.27, *p* = 0.044). Lung‐kidney transplantation was not significantly associated with graft failure across age groups. Given the small number of events in several MOLT subgroups, these age‐ and era‐specific findings should be interpreted as exploratory.

Sensitivity analyses supported the primary findings. In a landmark analysis restricted to 1‐year survivors, the apparent survival advantage observed in the post‐2020 era was attenuated and no longer statistically significant, suggesting that the observed survival advantage was influenced by improved early post‐transplant outcomes together with shorter available follow‐up. Similarly, adjustment for transplant center volume did not materially alter the overall findings, although higher‐volume centers were independently associated with improved graft survival (Supporting Information).

### 3.7 Cause of Death

Among 30 387 unique decedents, 32 931 cause‐of‐death records were identified. Because primary, secondary, and tertiary cause fields were treated equivalently, cause categories were not mutually exclusive. Using unique decedents as the denominator, the most commonly recorded causes were chronic rejection (14.3%, *n* = 4346), respiratory failure (13.8%, *n* = 4183), bacterial septicemia (6.0%, *n* = 1831), multiple organ failure (5.2%, *n* = 1589), and bacterial pneumonia (5.0%, *n* = 1502). Malignancy‐related causes were recorded for 6.3% of decedents, including metastatic malignancy (4.4%) and other malignancies (1.9%). COVID‐19 was recorded for 2.4% of decedents (*n* = 726), with all COVID‐19‐related deaths occurring from 2020 onward (Figure 4).

**FIGURE 4:**
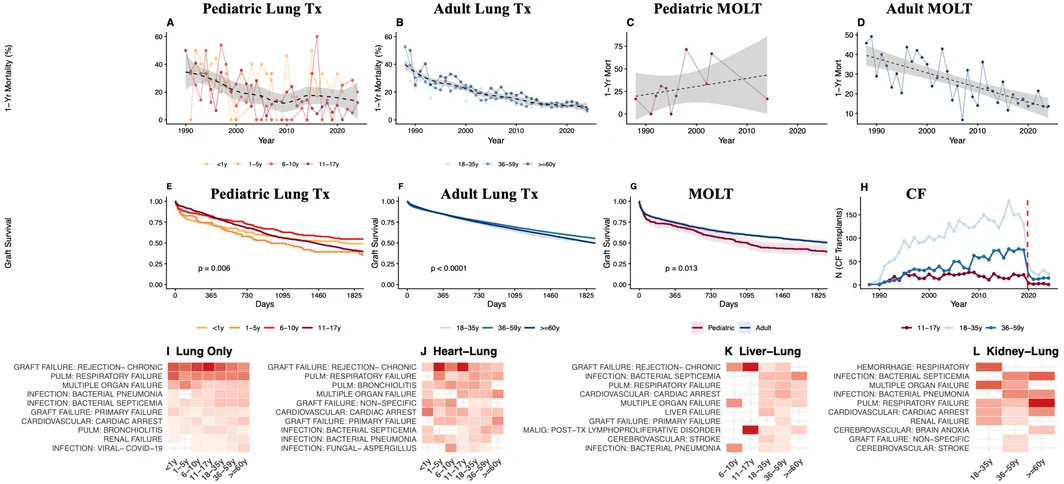
Longitudinal outcomes, graft survival, cystic fibrosis transplant volume, and cause‐of‐death profiles. Panels A–D: Annual 1‐year mortality trends. Annual 1‐year mortality rates for pediatric (A) and adult (B) isolated lung recipients and pediatric (C) and adult (D) multiorgan recipients. Points represent annual mortality rates, colored lines represent age‐specific trends, and black dashed lines represent cohort‐wide LOESS‐smoothed trends with 95% confidence intervals shown by gray ribbons. Panels E–G: Five‐year graft survival. Kaplan–Meier graft‐survival curves for pediatric isolated lung (E), adult isolated lung (F), and composite multiorgan (G) recipients. Shaded regions in Panel G represent 95% confidence intervals. *p* values were derived using log‐rank tests. Panel H: Cystic fibrosis transplant volume. Annual transplant volume among recipients with cystic fibrosis, stratified by age group. The vertical red dashed line indicates the 2019 U.S. Food and Drug Administration approval of elexacaftor/tezacaftor/ivacaftor, followed by a marked decline in CF‐related transplant volume. Panels I–L: Cause‐of‐death profiles. Heatmaps display the 10 most commonly recorded cause‐of‐death categories for isolated lung (I), lung‐heart (J), lung‐liver (K), and lung‐kidney (L) recipients. Primary, secondary, and tertiary cause‐of‐death fields were treated equivalently; therefore, categories were not mutually exclusive. The color gradient from light to dark red represents the percentage of unique decedents within each age stratum for whom each cause was recorded.

Cause‐of‐death profiles varied by transplant type. Lung‐heart recipients demonstrated higher rates of bronchiolitis‐related death (7.2% vs. 1.9% for isolated lung) and primary graft failure (5.2% vs. 2.5%). Lung‐liver recipients uniquely experienced liver failure‐related mortality (4.8%) and higher rates of post‐transplant lymphoproliferative disorder (PTLD; 4.8% vs. < 2% for isolated LTx). Lung‐kidney recipients had the highest rate of bacterial septicemia‐related death (10.9% vs. 6.0% for isolated LTx).

## 4 Discussion

The key finding of this study is that pediatric MOLT has evolved substantially alongside broader changes in the national lung transplant population. Historically, lung‐heart transplantation accounted for the majority of MOLT procedures, particularly among younger recipients, but its relative contribution declined over time. In contrast, lung‐liver and lung‐kidney transplantation remained uncommon but became increasingly established among adults. These procedural changes occurred as lung transplantation increasingly shifted toward older recipients, while pediatric and adolescent transplant volumes declined [10, 11, 12, 13]. Following the widespread availability of highly effective CFTR modulator therapy, transplantation for CF decreased markedly, and pediatric indications increasingly reflected pulmonary vascular and congenital disorders, whereas restrictive lung diseases such as idiopathic pulmonary fibrosis predominated among older adults [2, 10, 11, 12, 13, 14, 15].

Lung‐liver and lung‐kidney transplantation followed distinct age‐ and diagnosis‐specific trajectories. Lung‐liver transplantation remained most closely associated with CF, with idiopathic pulmonary fibrosis also contributing substantially among adults, whereas lung‐kidney transplantation was concentrated almost entirely among adults with CF and other fibrotic lung diseases. Their increasing use likely reflects the growing number of candidates with advanced pulmonary disease and clinically significant extrapulmonary organ dysfunction. In contrast, pediatric lung‐liver transplantation remained rare and pediatric lung‐kidney transplantation was nearly absent, limiting statistical precision and precluding robust evaluation of center‐level variation [10]. Standardized simultaneous lung‐kidney allocation criteria implemented in 2023 further reflect increasing recognition of kidney dysfunction in contemporary lung transplant candidates [16, 17]. Despite increasing procedural complexity and recipient acuity, improvements in graft survival and early outcomes occurred alongside advances in surgical technique, perioperative care, and candidate selection [11, 12].

Increasing candidate acuity provides important context for contemporary transplantation trends in the United States. ECMO support at transplantation increased substantially across adult age groups and among adolescents, paralleling national experience with the expanding use of ECMO as a bridge to transplantation [18, 19, 20]. Mechanical ventilation remained common among infants and increased modestly among adults. These findings demonstrate that transplant programs increasingly manage recipients requiring advanced life support at transplantation. For carefully selected candidates with concurrent end‐stage lung disease and dysfunction of another solid organ, combined transplantation may represent an appropriate strategy at centers with expertise in the perioperative and post‐transplant management of MOLT recipients.

Ischemic time increased in modern adult cohorts, coinciding with allocation‐policy changes that enabled broader donor sharing and wider geographic procurement, as well as expanded donor utilization, including donation after circulatory death and ex vivo lung perfusion [11]. Although the individual contributions of these factors could not be evaluated in the registry, differences in ischemic time across transplant configurations underscore that comparisons between isolated LTx and MOLT reflect both recipient characteristics and procedural logistics.

Overall outcomes after MOLT were broadly comparable to those after isolated lung transplantation, although associations varied by organ combination, transplant era, and recipient age. Graft‐survival gains after isolated LTx mirrored previously published reports [11, 12]. Competing‐risks modeling attenuated the apparent age‐associated hazard, highlighting the contribution of non‐graft‐related mortality among older recipients. This distinction is particularly relevant in contemporary adult practice, where competing mortality may increase even as graft‐related outcomes improve. Lung‐liver transplantation was associated with lower adjusted graft‐failure risk overall, whereas lung‐heart and lung‐kidney transplantation showed no consistent overall advantage or disadvantage. Prior studies have similarly reported survival after lung‐liver transplantation comparable to isolated LTx in selected cohorts and acceptable outcomes after lung‐kidney transplantation under structured eligibility criteria [16, 17]. However, findings from pediatric recipients and other small MOLT subgroups should be interpreted cautiously. The apparent survival advantage in the 2020–2024 era was attenuated in landmark analyses, suggesting contributions from improved early outcomes and shorter available follow‐up. Primary graft dysfunction appeared uncommon in registry coding, although the low reported rates likely reflect incomplete registry capture rather than the true incidence of early graft injury.

This study has several important limitations. As a retrospective registry analysis, the findings are subject to residual confounding, selection bias, and changes in data availability and reporting across eras. Several MOLT subgroups, particularly lung‐liver, lung‐kidney, other multiorgan configurations, and pediatric recipients, were small, limiting statistical precision and rendering subgroup analyses exploratory. Follow‐up was shorter for recipients transplanted during 2020–2024, limiting interpretation of long‐term outcomes. The proportional hazards assumption was violated, indicating that some associations varied over time; hazard ratios should therefore be interpreted cautiously. Post‐transplant complications were analyzed as time‐fixed indicators of occurrence during follow‐up, so these exploratory associations are susceptible to temporal‐order and immortal‐time bias and should not be interpreted as causal. Cause‐of‐death categories were derived from multiple non‐mutually‐exclusive fields. Finally, the UNOS registry lacks detailed information regarding center‐specific practices, surgeon experience, immunosuppressive strategies, and contemporary CLAD phenotypes, and the availability of variables such as cPRA changed over time.

In summary, pediatric MOLT evolved substantially over the study period, with declining use of lung‐heart transplantation, changing diagnostic indications, and persistently limited use of lung‐liver and lung‐kidney transplantation. The expansion of lung‐liver and lung‐kidney transplantation among adults provides important national context for understanding these pediatric trends. Although early outcomes improved across transplant eras, the small number of pediatric and other uncommon MOLT procedures warrants cautious interpretation of subgroup findings. This national registry analysis provides a foundation for prospective multicenter studies of recipient selection and long‐term outcomes in pediatric MOLT.

## Funding

This study received no external funding. The work was supported by institutional resources.

## Disclosure

The data reported here have been supplied by the United Network for Organ Sharing as the contractor for the Organ Procurement and Transplantation Network. The interpretation and reporting of these data are the responsibility of the author(s) and in no way should be seen as an official policy of or interpretation by the OPTN or the U.S. Government.

## Conflicts of Interest

The authors declare no conflicts of interest relevant to this study.

## Supporting information


**Figure S1:** Study flow diagram and cohort selection criteria. Among 227 432 thoracic transplant candidates identified from 1987 to 2024, 73 709 did not undergo transplantation, leaving 153 723 thoracic transplant recipients. The heart‐involved (*n* = 98 549) and lung‐involved (*n* = 56 760) groups were descriptive, non‐mutually‐exclusive classifications; 1 586 recipients underwent procedures involving both the heart and lungs. The lung‐involved study cohort comprised 54 797 isolated lung transplant recipients and 1 963 MOLT recipients, classified into mutually exclusive groups of dual‐organ lung‐heart (*n* = 1557), lung‐liver (*n* = 216), lung‐kidney (*n* = 154), and other multiorgan configurations (*n* = 36). In the diagram, the box labeled “Triple+ Organ” represents the residual group of other multiorgan configurations, including higher‐order procedures and lung‐pancreas transplantation.
**Figure S2:** Procedural Distribution and Diagnostic Variance Across Study Eras. Panel A: Transplant Type Distribution by Era. Stacked bar charts depict the longitudinal distribution of transplant procedures—isolated lung, lung‐heart, lung‐liver, and lung‐kidney—stratified by age group and transplant era. The data demonstrate the persistent role of multiorgan transplantation in pediatric populations and the marked expansion of dual‐organ procedures among adult and senior recipients in the modern era. Panel B: Diagnostic Variance (Unique Indications per Era). Bar charts display the total number of unique primary diagnostic codes recorded per study era within each age stratum. The progressive increase in diagnostic diversity across most cohorts reflects expanding clinical indications, improved diagnostic granularity within the UNOS registry, and the growing management of heterogeneous and complex pulmonary disease phenotypes over time. This supplemental figure complements Figure 1 and Figure 2 by illustrating that, although a limited number of diagnoses account for the majority of transplant volume (e.g., IPF and COPD among senior recipients), the field has evolved toward greater procedural diversity (Panel C) and increasing diagnostic complexity (Panel D).
**Figure S3:** Temporal trends in transplantation volume by age, indication, and organ combination (1987–2024). Panel A: Relative proportion of recipient age groups. Annual transplant volume stratified by age and faceted into pediatric (< 18 years) and adult (≥ 18 years) cohorts. Trends demonstrate increasing predominance of senior recipients (≥ 60 years) and shifting distributions within pediatric age categories. Panel B: Proportional shift in age categories. Longitudinal comparison of adult and pediatric transplants expressed as a percentage of total annual transplant volume, illustrating the progressive transition toward adult‐oriented transplantation. Panel C: Impact of clinical advances on cystic fibrosis (CF) volume. Annual number of transplants performed for a primary diagnosis of CF. The vertical dashed line (2019.8) denotes the introduction of highly effective cystic fibrosis transmembrane conductance regulator (CFTR) modulator therapy (elexacaftor‐tezacaftor‐ivacaftor), corresponding with a marked decline in CF‐related transplant volume. Panel D: Volume growth by organ system. Faceted area charts display total annual transplant volumes for isolated lung, lung‐heart, lung‐liver, and lung‐kidney procedures. Independent y‐axis scales highlight differences in magnitude and the relative growth and stabilization of multiorgan programs compared with isolated lung transplantation.
**Table S1:** Characteristics of isolated lung transplantation by age group and era.

## Data Availability

The data that support the findings of this study are available from UNOS. Restrictions apply to the availability of these data, which were used under license for this study. Data are available from https://optn.transplant.hrsa.gov/data/view‐data‐reports/request‐data/ with the permission of UNOS.
