# Supplementary material for: Pediatric Multiorgan Lung Transplantation: Four Decades of National Trends and Outcomes
Source: Pediatr Transplant. 2026 Oct 15;30(10):e70466. doi: 10.1111/petr.70466 (PMC13673004; doi:10.1111/petr.70466)
Supplement: Supplementary file 1 [file PETR-30-e70466-s001.docx]

**Supplementary Methods**

**Definition of Graft Failure**

Graft failure was defined as the earliest occurrence of: (1) retransplantation, (2) death from any cause, or (3) documented graft failure, coded as GSTATUS = 1 in the UNOS database.

**Visualization of Temporal Trends**

Temporal trends were visualized using area charts, line graphs, and stacked bar charts stratified by age group and transplant era.

**Statistical Analysis**

Continuous variables were summarized as mean ± standard deviation for normally distributed data or median (interquartile range) for skewed distributions. Categorical variables were summarized as counts and percentages. Graft survival was estimated using Kaplan–Meier methods, with 1-, 5-, and 10-year survival probabilities reported with 95% confidence intervals. Survival curves were stratified by transplant era and age group, and differences were assessed using log-rank tests.

Multivariable Cox proportional hazards regression was used to identify independent predictors of graft failure among isolated lung transplant recipients, with results reported as hazard ratios (HRs) and 95% confidence intervals. The primary model included transplant era, age group, primary diagnosis category, pretransplant mechanical ventilation, pretransplant ECMO, and ischemic time (modeled as a continuous variable). Model discrimination was assessed using Harrell’s C-index. Given the clinical heterogeneity and large sample size, violations of the proportional hazards assumption were anticipated and results were interpreted cautiously, acknowledging potential time-varying effects. Age-stratified Cox regression models were used to evaluate whether the effects of key risk factors (era, diagnosis, mechanical ventilation, ECMO, and ischemic time) differed across the seven physiologic age groups.

To compare isolated lung and lung-based multiorgan transplant recipients, unadjusted and adjusted Cox models were fitted with transplant type (isolated lung, lung-heart, lung-liver, lung-kidney) as the primary exposure. Adjusted models controlled for age group, transplant era, primary diagnosis, pretransplant mechanical ventilation, and ECMO support. Interactions between transplant type and transplant era, as well as between transplant type and age group, were evaluated using likelihood ratio tests.

Competing risks of death with a functioning graft were addressed using Fine–Gray subdistribution hazard regression to model the cumulative incidence of graft failure. Models included baseline covariates from the primary Cox model; post-transplant complications were excluded. Subdistribution hazard ratios (SHRs) with 95% confidence intervals were reported, and cumulative incidence curves were stratified by transplant era.

A landmark analysis restricted to recipients surviving to 1 year post-transplant was performed to evaluate conditional graft survival, using Cox regression beginning at the 1-year landmark and including the same covariates as the primary model. Formal interaction testing was conducted to assess effect modification by age group and transplant era, with significance evaluated using likelihood ratio tests.

All analyses were performed using R version 4.5.2 (R Foundation for Statistical Computing, Vienna, Austria), with packages including *survival* and *tidycmprsk*. No adjustment was made for multiple comparisons given the exploratory nature of several analyses. Details regarding missing data handling and sensitivity analyses, including approaches to mitigate immortal time bias, are provided in the Supplementary Materials.

**Survival Analysis**

Survival curves were stratified by transplant era and age group. Differences between groups were assessed using log-rank tests.

Transplant centers were categorized according to annual transplant volume and classified as low-volume (<15 transplants per year), medium-volume (15–30 transplants per year), or high-volume (>30 transplants per year).

Multivariable Cox proportional hazards regression models included transplant era, age group, primary diagnosis category, pretransplant mechanical ventilation, pretransplant extracorporeal membrane oxygenation (ECMO) support, and ischemic time (modeled as a continuous variable in hours). The proportional hazards assumption was assessed using Schoenfeld residuals and formal statistical testing with the *cox.zph* function.

**Cause-of-Death Classification**

Deaths were classified using UNOS standardized cause-of-death codes and mapped to clinically meaningful categories, including chronic rejection, respiratory failure, infection (bacterial, viral, or fungal), malignancy, cardiovascular causes, multiple organ failure, and other specified causes. A standardized cause-of-death lookup table was merged with death records to generate human-readable categories.

Cause-of-death distributions were tabulated overall and stratified by: (1) age group (pediatric vs adult), (2) transplant type (isolated lung, lung-heart, lung-liver, lung-kidney), (3) transplant era, and (4) time since transplantation. For time-dependent analyses, deaths were classified as early (<1 year), intermediate (1–5 years), or late (>5 years) post-transplant. The top 5–10 causes within each stratum were reported as counts and percentages.

**Software and Reproducibility**

Survival analyses were conducted using the *survival* package (version 3.5-5). Competing risks analyses utilized the *tidycmprsk* package (version 1.0.0). Data manipulation was performed using *dplyr* (version 1.1.2) and *tidyr* (version 1.3.0). Visualizations were generated using *ggplot2* (version 3.4.2) and *ggsurvfit* (version 1.0.0). Statistical significance was defined as a two-sided p value <0.05. No adjustments were made for multiple comparisons given the exploratory nature of several analyses.

**Missing Data**

Patterns of missing data were assessed for all key variables. Recipients with missing survival time or vital status were excluded from survival analyses (n=836, 1.5%). Complete-case analysis was used for multivariable models, with analytic sample sizes varying by model; missing ischemic time (n=2,028) was the most common reason for exclusion. No imputation was performed because of the large sample size and low overall missingness for most variables. Model-specific sample sizes are reported with the corresponding analyses.

**Sensitivity Analyses**

Given the potential for immortal time bias and paradoxical associations between post-transplant complications (malignancy, diabetes mellitus, and dialysis dependence) and graft survival in standard Cox models, these variables were excluded from Fine–Gray competing risks models. This approach allowed competing risks analyses to focus on baseline predictors of graft failure. Attenuation of sex- and age-related effects in competing risks models was interpreted as evidence of competing mortality rather than graft-specific mechanisms.

**Supplemental Figures and Results**

**Supplementary Figure 1. Study flow diagram and cohort selection criteria.** Among 227,432 thoracic transplant candidates identified from 1987–2024, 73,709 did not undergo transplantation, leaving 153,723 thoracic transplant recipients. The heart-involved (n=98,549) and lung-involved (n=56,760) groups were descriptive, non-mutually-exclusive classifications; 1,586 recipients underwent procedures involving both the heart and lungs. The lung-involved study cohort comprised 54,797 isolated lung transplant recipients and 1,963 MOLT recipients, classified into mutually exclusive groups of dual-organ lung-heart (n=1,557), lung-liver (n=216), lung-kidney (n=154), and other multiorgan configurations (n=36). In the diagram, the box labeled “Triple+ Organ” represents the residual group of other multiorgan configurations, including higher-order procedures and lung-pancreas transplantation.

**Primary Diagnoses by Age Group and Transplant Type**

**Isolated Lung Recipients**

The diagnostic profile for isolated lung recipients demonstrated a pronounced age-related shift, from congenital and pulmonary vascular conditions in infancy to obstructive and restrictive lung diseases in older adults. Infants (<1 year) were primarily transplanted for pulmonary hypertension or PAH (n=29) and pulmonary vascular disease (n=20). Within the pediatric population (1–17 years), cystic fibrosis was the predominant indication, totaling 655 cases across age groups (1–5 years: n=5; 6–10 years: n=96; 11–17 years: n=554). This dominance persisted into young adulthood (18–35 years), where cystic fibrosis remained the leading indication (n=3,438). Middle-aged adults (36–59 years) transitioned toward COPD or emphysema (n=6,001). Senior recipients (≥60 years) were overwhelmingly driven by idiopathic pulmonary fibrosis (IPF; n=10,757) and COPD or emphysema (n=7,504).

**Lung-Heart Recipients**

Indications for combined lung-heart transplantation were largely concentrated in pulmonary vascular disease and congenital cardiac conditions (Figure 1D). In the pediatric cohort (<18 years), the leading indications were pulmonary hypertension or PAH (n=68) and congenital heart defects with unknown prior surgical status (n=22). Among adults (≥18 years), PAH predominated (n=325), followed by Eisenmenger syndrome secondary to ventricular septal defect (n=118).

**Lung-Liver Recipients**

Combined lung-liver transplantation demonstrated a diagnostic focus on cystic fibrosis and restrictive lung diseases. In adults (≥18 years), cystic fibrosis was the leading indication (n=75), followed by IPF (n=52). The pediatric lung-liver cohort (<18 years) was similarly led by cystic fibrosis (n=16).

**Lung-Kidney Recipients**

The lung-kidney cohort was characterized by systemic, multiorgan disease processes. Among adults (≥18 years), the most common indications were cystic fibrosis (n=20) and pulmonary fibrosis from other specified causes (n=19). Lung-kidney transplantation in pediatric patients (<18 years) was exceedingly rare, with a single case performed for lung re-transplantation due to obliterative bronchiolitis (n=1).

**Mechanical Ventilation Trends by Era.**

Infants (<1 year) consistently demonstrated the highest reliance on pre-transplant mechanical ventilation across all eras, with rates of 56.5% (n=35) in Era 1 (1987-1999), 73.8% (n=45) in Era 2 (2000–2009), 66.1% (n=39) in Era 3 (2010–2019), and 70.0% (n=7) in Era 4 (2020-2024). In contrast, senior recipients (≥60 years) exhibited minimal ventilator dependence, with the lowest observed rate of 1.2% (n=12) in the <2000 era. A temporal increase in pre-transplant ventilation was observed among young adults (18–35 years), rising from 5.5% (<2000) to 16.5% (>2020), and among middle-aged adults (36–59 years), increasing from 2.7% to 9.3% over the same period.

**A**

**B**

**Supplemental Figure 2. Procedural Distribution and Diagnostic Variance Across Study Eras.**

**Panel A: Transplant Type Distribution by Era.** Stacked bar charts depict the longitudinal distribution of transplant procedures—isolated lung, lung-heart, lung-liver, and lung-kidney—stratified by age group and transplant era. The data demonstrate the persistent role of multiorgan transplantation in pediatric populations and the marked expansion of dual-organ procedures among adult and senior recipients in the modern era.

**Panel B: Diagnostic Variance (Unique Indications per Era).** Bar charts display the total number of unique primary diagnostic codes recorded per study era within each age stratum. The progressive increase in diagnostic diversity across most cohorts reflects expanding clinical indications, improved diagnostic granularity within the UNOS registry, and the growing management of heterogeneous and complex pulmonary disease phenotypes over time.

This supplemental figure complements Figures 1 and 2 by illustrating that, although a limited number of diagnoses account for the majority of transplant volume (e.g., IPF and COPD among senior recipients), the field has evolved toward greater procedural diversity (Panel C) and increasing diagnostic complexity (Panel D).

**Supplemental Figure 3. Temporal trends in transplantation volume by age, indication, and organ combination (1987-2024).**

**Panel A: Relative proportion of recipient age groups. Annual** transplant volume stratified by age and faceted into pediatric (<18 years) and adult (≥18 years) cohorts. Trends demonstrate increasing predominance of senior recipients (≥60 years) and shifting distributions within pediatric age categories.

**Panel B: Proportional shift in age categories.** Longitudinal comparison of adult and pediatric transplants expressed as a percentage of total annual transplant volume, illustrating the progressive transition toward adult-oriented transplantation.

**Panel C: Impact of clinical advances on cystic fibrosis (CF) volume.** Annual number of transplants performed for a primary diagnosis of CF. The vertical dashed line (2019.8) denotes the introduction of highly effective cystic fibrosis transmembrane conductance regulator (CFTR) modulator therapy (elexacaftor-tezacaftor-ivacaftor), corresponding with a marked decline in CF-related transplant volume.

**Panel D: Volume growth by organ system.** Faceted area charts display total annual transplant volumes for isolated lung, lung-heart, lung-liver, and lung-kidney procedures. Independent y-axis scales highlight differences in magnitude and the relative growth and stabilization of multiorgan programs compared with isolated lung transplantation.

**Graft Failure**

The incidence of immediate post-operative graft failure, specifically primary graft dysfunction (PGD), declined substantially over time across nearly all adult age groups despite increasing recipient clinical complexity. Among senior recipients (≥60 years), PGD peaked at 4.1% during the 2000–2019 era and improved to 0.9% in the modern era (>2020). A similar pattern was observed in middle-aged adults (36–59 years), with PGD decreasing from a peak of 4.6% in 2000–2009 to 1.3% after 2020.

In pediatric cohorts, temporal patterns were more variable. Infants (<1 year) experienced a marked increase in PGD during the 2010–2019 era, reaching 10.2% (n=6), followed by complete resolution (0%) in the current era. Adolescents (11–17 years) demonstrated a comparable trajectory, with PGD peaking at 5.0% (n=19) in 2000–2009 and subsequently declining to 0%.

Early graft failure attributable to acute rejection remained consistently uncommon across most eras and age groups, generally below 2%. A notable exception occurred among adolescents (11–17 years) during the 2010–2019 era, when rejection-related early graft failure increased to 5.1% (n=14). Overall 1-year graft failure rates, serving as a composite indicator of early post-transplant success, improved markedly over time. In the earliest era (<2000), 1-year graft failure occurred in 33.9% of infants and 30.1% of senior recipients. By the modern era (>2020), these rates declined to 6.2% among adolescents and 10.0% among seniors.

**Chronic Rejection.**

Chronic rejection, predominantly manifesting as chronic lung allograft dysfunction (CLAD), represented the principal cause of long-term graft loss across the cohort. Unlike immediate surgical failure, chronic rejection remained disproportionately prevalent among younger recipients. Adolescents aged 11–17 years experienced chronic rejection in 22.2% of cases, comparable to the 21.7% observed among children aged 6–10 years. Rates decreased progressively with advancing age, affecting 15.6% of young adults (18–35 years), 10.4% of middle-aged adults (36–59 years), and 7.5% of senior recipients (≥60 years).

**Metabolic, Renal, and Oncologic Morbidity.**

Metabolic complications varied by age and era. New-onset diabetes occurred in 11.7% (n=32) of adolescents during the 2010–2019 era.

Pediatric malignancy rates ranged from 0% to 16.7% across age-by-era strata; the highest estimate represented one case among six recipients aged 1–5 years in 2020–2024.

**Hospital Length of Stay and Cognitive Outcomes.**

Infants demonstrated the longest hospitalizations overall, with median stays of 70 days (IQR 52–103) for lung-heart procedures and 51 days (IQR 33–80) for isolated lung transplantation.

Neurodevelopmental and cognitive outcomes displayed pronounced age-related differences. While 82% of young adults returned to normal functional status post-transplant, significant cognitive impairment was observed among the youngest recipients. Definite cognitive delay or impairment was documented in 21.1% (n=278) of infants and 6.9% (n=38) of children aged 1–5 years following isolated lung transplantation. Corresponding rates were lower among lung-heart recipients, affecting 10.6% (n=11) of infants and 5.2% (n=10) of children aged 1–5 years.

**Predictors of Survival and Graft Failure**

In multivariable Cox regression, graft survival improved progressively across transplant eras. Relative to the pre-2000 era, adjusted hazard ratios demonstrated significant risk reductions in 2000–2009 (HR 0.83, 95% CI 0.80–0.86), 2010–2019 (HR 0.64, 95% CI 0.62–0.66), and >2020 (HR 0.53, 95% CI 0.50–0.56; all p<0.001), corresponding to 17%, 36%, and 47% reductions in graft failure risk, respectively.

Age demonstrated a non-linear association with graft failure. Relative to infants (<1 year), young children (1–5 and 6–10 years) showed no significant difference in risk (p>0.05), whereas adolescents (11–17 years) exhibited the highest risk among pediatric recipients (HR 1.77, 95% CI 1.45–2.14, p<0.001). Among adults, risk increased progressively with age: 18–35 years (HR 1.37, 95% CI 1.14–1.64), 36–59 years (HR 1.47, 95% CI 1.23–1.76), and ≥60 years (HR 2.06, 95% CI 1.72–2.46; all p<0.001).

Temporal improvements varied by age group. Adults aged 36–59 years (HR 0.64, 95% CI 0.58–0.71) and ≥60 years (HR 0.62, 95% CI 0.57–0.68) derived the greatest benefit in the >2020 era (both p<0.001). In contrast, infants showed no significant era-related improvement (all p>0.22). Children aged 6–10 years demonstrated substantial improvement in the 2010–2019 era (HR 0.37, 95% CI 0.23–0.62, p<0.001). Adolescents showed benefit in 2010–2019 (HR 0.70, 95% CI 0.57–0.87, p=0.002), but this did not persist in the >2020 era (HR 0.63, p=0.145).

Markers of pre-transplant severity were strong predictors of graft failure. Mechanical ventilation was associated with increased risk (HR 1.24, 95% CI 1.18–1.31, p<0.001) and remained harmful across most age groups, including adolescents (HR 1.59), young adults (HR 1.31), middle-aged adults (HR 1.21), and seniors (HR 1.22; all p<0.001), but not in the youngest children. ECMO support was similarly associated with increased risk overall (HR 1.21, 95% CI 1.13–1.30, p<0.001) but demonstrated age-dependent effects, conferring excess risk only among adults aged 36–59 years (HR 1.20) and ≥60 years (HR 1.31; both p<0.001), with no significant effect in pediatric cohorts.

Diagnosis-specific differences were modest. Compared with cystic fibrosis, IPF/ILD showed marginally increased risk (HR 1.04, 95% CI 1.01–1.07, p=0.010). Model assumptions were violated (global p<2×10⁻¹⁶), indicating that the effects of some variables changed over time, particularly transplant era; hazard ratios should therefore be interpreted cautiously.

**Cause of Death**

Chronic rejection was substantially more prevalent among pediatric deaths compared with adults (25.0% vs 14.2%), with the highest proportion observed in adolescents (26.6%). Respiratory failure occurred at similar rates in pediatrics and adults (15.6% vs 14.0%). Multiple organ failure was more frequent in very young children (<1 year: 8.7%; 1–5 years: 13.2%) compared with adults (5.1%). Malignancy-related deaths were nearly absent in pediatric recipients (0% vs 4.6% in adults). COVID-19–related and cardiovascular deaths occurred almost exclusively in adults. Infection-related mortality differed by age, with bacterial septicemia (3.7% vs 6.1%) and bacterial pneumonia (3.0% vs 5.0%) occurring less frequently in pediatric recipients, while fungal aspergillosis was observed uniquely among children aged 1–5 years (7.5%).

Temporal shifts in causes of death were evident across eras. Chronic rejection was the leading cause of death in the pre-2000 (14.0%) and 2000–2009 (15.9%) eras. During 2010–2019, respiratory failure became the leading cause (16.3%), surpassing chronic rejection (14.7%), while malignancy emerged as the fourth leading cause (5.5%). Among recipients transplanted in 2010–2019, COVID-19 ranked as the fifth most commonly recorded cause of death (5.3%); these deaths occurred after 2020 during subsequent follow-up. In the >2020 era, chronic rejection declined to the fifth leading cause (6.5%), whereas multiple organ failure rose to second (9.0%) and bacterial septicemia to third (8.0%).

When stratified by time from transplant, early mortality (<1 year; n=7,887 deaths) was dominated by bacterial septicemia (11.6%), respiratory failure (9.7%), multiple organ failure (9.4%), and primary graft failure (6.2%). In the intermediate period (1–5 years; n=11,994 deaths), chronic rejection predominated (21.3%), followed by respiratory failure (18.2%), with emerging malignancy-related mortality (4.7%). In the late period (>5 years; n=10,112 deaths), chronic rejection remained the leading cause (18.7%), while metastatic malignancy rose to the third leading cause (7.8%), nearly doubling from the intermediate period, and respiratory failure accounted for 15.5%.

**Competing Risks Analysis**

Fine–Gray competing risks models (N=53,962) accounting for death with a functioning graft (n=781, 1.4%) confirmed progressive temporal improvements in graft outcomes. Era effects were slightly stronger than those observed in Cox models, with subdistribution hazard ratios of 0.79 (95% CI 0.77–0.82) for 2000–2009, 0.66 (95% CI 0.63–0.68) for 2010–2019, and 0.58 (95% CI 0.55–0.62) for >2020 (all p<0.001). Correspondingly, the cumulative incidence of graft failure declined substantially over time: 1-year incidence decreased from 26.1% in the pre-2000 era to 12.0% in the >2020 era, while 5-year incidence declined from 56.3% in the earliest era to 42.5% in 2010–2019. Among patients with sufficient follow-up, 10-year cumulative incidence ranged from 70.2% to 76.2%.

Compared with Cox models, competing risks analyses attenuated age-related effects, particularly in cohorts with higher competing mortality. Adolescents demonstrated SHR 1.55 (95% CI 1.21–1.97, p<0.001) compared with Cox HR 1.77, while senior recipients showed SHR 1.63 (95% CI 1.29–2.05, p<0.001) compared with Cox HR 2.06. Young children remained non-significant in both frameworks (1–5 years: SHR 1.30, p=0.210; 6–10 years: SHR 1.14, p=0.360).

Pre-transplant severity markers remained robust predictors, with mechanical ventilation (SHR 1.26, 95% CI 1.19–1.34, p<0.001) and ECMO support (SHR 1.22, 95% CI 1.13–1.32, p<0.001) showing effect sizes comparable to Cox models. In contrast, sex differences were no longer statistically significant (SHR 1.02, p=0.070), suggesting that the excess male risk observed in Cox models was partially driven by competing mortality rather than graft-specific mechanisms. Diagnosis effects remained modest (IPF/ILD SHR 1.03, p=0.023).

**Center Volume Effects and Interaction Analyses**

Transplant centers were categorized by annual volume as low (<15 transplants/year), medium (15–30/year), or high (>30/year). After adjustment for era, age, diagnosis, mechanical ventilation, and ECMO, high-volume centers demonstrated a 13% lower risk of graft failure compared with low-volume centers (HR 0.87, 95% CI 0.84–0.90, p<0.001), while medium-volume centers demonstrated a 7% reduction (HR 0.93, 95% CI 0.89–0.96, p<0.001). A significant volume-by-era interaction (p=0.009) indicated that center volume effects strengthened over time.

Among 44,847 recipients surviving beyond one year, conditional survival analyses demonstrated persistent but attenuated era effects: 2000–2009 HR 0.88 (p<0.001) and 2010–2019 HR 0.84 (p<0.001), while the >2020 era no longer demonstrated a significant benefit (HR 0.96, p=0.29), likely reflecting limited long-term follow-up. These findings suggest that the apparent survival advantage in the 2020–2024 era was influenced by improved early post-transplant outcomes and limited longer-term follow-up. The age-by-era interaction approached but did not reach statistical significance (p=0.10), suggesting that the adolescent survival disadvantage has narrowed but not fully resolved. The significant volume-by-era interaction (p=0.009) further confirms the increasing importance of center experience in modern lung transplantation.

**Supplemental Table 1.** Characteristics of isolated lung transplantation by age group and era

| **Parameter** | **AgeGroup_2** | **<1987-1999** | **2000-2009** | **2010-2019** | **2020-2024** |
| --- | --- | --- | --- | --- | --- |
| **N** | **<1y** | 62 | 61 | 59 | 10 |
|  | **1-5y** | 33 | 28 | 20 | 6 |
|  | **6-10y** | 79 | 83 | 61 | 17 |
|  | **11-17y** | 258 | 379 | 274 | 64 |
|  | **18-35y** | 1292 | 1900 | 2135 | 563 |
|  | **36-59y** | 4274 | 7007 | 7612 | 3631 |
|  | **>=60y** | 1038 | 4997 | 11711 | 7143 |
| **Re-transplantation (%)** | **<1y** | 3 (4.8%) | 1 (1.6%) | 0 (0%) | 0 (0%) |
|  | **1-5y** | 5 (15.2%) | 4 (14.3%) | 2 (10%) | 0 (0%) |
|  | **6-10y** | 13 (16.5%) | 5 (6%) | 7 (11.5%) | 1 (5.9%) |
|  | **11-17y** | 19 (7.4%) | 33 (8.7%) | 12 (4.4%) | 4 (6.2%) |
|  | **18-35y** | 52 (4%) | 155 (8.2%) | 258 (12.1%) | 76 (13.5%) |
|  | **36-59y** | 129 (3%) | 296 (4.2%) | 352 (4.6%) | 209 (5.8%) |
|  | **>=60y** | 11 (1.1%) | 143 (2.9%) | 227 (1.9%) | 104 (1.5%) |
| **Waitlist time, days (median [IQR])** | **<1y** | 20 (7-44) | 52 (30-89) | 52 (28-86) | 82 (51-103) |
|  | **1-5y** | 58 (34-145) | 63 (18-130) | 53 (29-121) | 107 (66-152) |
|  | **6-10y** | 153 (60-328) | 193 (53-347) | 121 (69-238) | 124 (36-169) |
|  | **11-17y** | 228 (97-429) | 173 (49-539) | 45 (15-157) | 36 (11-82) |
|  | **18-35y** | 258 (96-493) | 179 (48-586) | 56 (16-176) | 32 (10-99) |
|  | **36-59y** | 234 (96-462) | 167 (47-482) | 56 (16-178) | 27 (9-82) |
|  | **>=60y** | 245 (98-444) | 94 (26-300) | 49 (14-148) | 31 (10-89) |
| **Pre-TX creatinine, mg/dL (mean ± SD)** | **<1y** | 0.44 ± 0.31 | 0.33 ± 0.23 | 0.42 ± 1.67 | 0.16 ± 0.03 |
|  | **1-5y** | 0.36 ± 0.15 | 0.34 ± 0.18 | 0.32 ± 0.17 | 0.25 ± 0.08 |
|  | **6-10y** | 0.48 ± 0.17 | 0.67 ± 1.74 | 0.34 ± 0.12 | 0.38 ± 0.17 |
|  | **11-17y** | 0.65 ± 0.31 | 0.69 ± 1.09 | 0.48 ± 0.2 | 0.54 ± 0.26 |
|  | **18-35y** | 0.91 ± 0.77 | 0.8 ± 0.57 | 0.73 ± 0.33 | 0.75 ± 0.38 |
|  | **36-59y** | 0.97 ± 1.39 | 0.9 ± 0.53 | 0.84 ± 0.39 | 0.81 ± 0.36 |
|  | **>=60y** | 1.12 ± 2.21 | 0.92 ± 0.43 | 0.87 ± 0.4 | 0.85 ± 0.28 |
| **Functionally disabled at TX (%)** | **<1y** | 56 (90.3%) | 60 (98.4%) | 59 (100%) | 10 (100%) |
|  | **1-5y** | 21 (63.6%) | 25 (89.3%) | 20 (100%) | 6 (100%) |
|  | **6-10y** | 38 (48.1%) | 55 (66.3%) | 61 (100%) | 17 (100%) |
|  | **11-17y** | 136 (52.7%) | 279 (73.6%) | 274 (100%) | 62 (96.9%) |
|  | **18-35y** | 623 (48.2%) | 225 (11.8%) | 23 (1.1%) | 24 (4.3%) |
|  | **36-59y** | 1744 (40.8%) | 648 (9.2%) | 89 (1.2%) | 96 (2.6%) |
|  | **>=60y** | 326 (31.4%) | 259 (5.2%) | 118 (1%) | 212 (3%) |
| **Ventilation before TX (%)** | **<1y** | 35 (56.5%) | 45 (73.8%) | 39 (66.1%) | 7 (70%) |
|  | **1-5y** | 5 (15.2%) | 11 (39.3%) | 5 (25%) | 5 (83.3%) |
|  | **6-10y** | 12 (15.2%) | 4 (4.8%) | 8 (13.1%) | 2 (11.8%) |
|  | **11-17y** | 32 (12.4%) | 28 (7.4%) | 32 (11.7%) | 11 (17.2%) |
|  | **18-35y** | 71 (5.5%) | 167 (8.8%) | 319 (14.9%) | 93 (16.5%) |
|  | **36-59y** | 115 (2.7%) | 283 (4%) | 604 (7.9%) | 337 (9.3%) |
|  | **>=60y** | 12 (1.2%) | 221 (4.4%) | 403 (3.4%) | 172 (2.4%) |
| **ECMO before TX (%)** | **<1y** | 4 (6.5%) | 2 (3.3%) | 9 (15.3%) | 2 (20%) |
|  | **1-5y** | 0 (0%) | 1 (3.6%) | 2 (10%) | 3 (50%) |
|  | **6-10y** | 4 (5.1%) | 0 (0%) | 5 (8.2%) | 3 (17.6%) |
|  | **11-17y** | 1 (0.4%) | 5 (1.3%) | 32 (11.7%) | 15 (23.4%) |
|  | **18-35y** | 7 (0.5%) | 29 (1.5%) | 282 (13.2%) | 150 (26.6%) |
|  | **36-59y** | 8 (0.2%) | 63 (0.9%) | 541 (7.1%) | 494 (13.6%) |
|  | **>=60y** | 0 (0%) | 30 (0.6%) | 288 (2.5%) | 243 (3.4%) |
| **Donor age, years (mean ± SD)** | **<1y** | 0.9 ± 1.4 | 0.5 ± 0.8 | 0.5 ± 0.9 | 0.2 ± 0.4 |
|  | **1-5y** | 4.4 ± 7.6 | 2.6 ± 2.1 | 2.3 ± 0.9 | 3.2 ± 2.1 |
|  | **6-10y** | 13.1 ± 13.8 | 8.7 ± 8.6 | 8 ± 4.6 | 12.8 ± 10.6 |
|  | **11-17y** | 21.6 ± 14.2 | 21 ± 13.5 | 18.9 ± 10.6 | 21.8 ± 11.4 |
|  | **18-35y** | 28.9 ± 12.6 | 30.8 ± 13.1 | 32.8 ± 12.8 | 33.1 ± 12.6 |
|  | **36-59y** | 29.7 ± 12.6 | 33.2 ± 14 | 35 ± 13.8 | 36.4 ± 13.1 |
|  | **>=60y** | 30.7 ± 13.4 | 34 ± 14.5 | 35.8 ± 14.1 | 37.6 ± 13.9 |
| **Distance traveled, miles (median [IQR])** | **<1y** | 402 (224-654) | 592 (234-706) | 601 (366-846) | 568 (335-830) |
|  | **1-5y** | 248 (90-605) | 494 (325-702) | 548 (374-702) | 326 (91-416) |
|  | **6-10y** | 283 (16-467) | 371 (190-614) | 525 (338-806) | 452 (340-736) |
|  | **11-17y** | 99 (2-306) | 175 (12-310) | 278 (132-453) | 378 (198-607) |
|  | **18-35y** | 84 (6-255) | 108 (14-294) | 139 (28-296) | 268 (120-570) |
|  | **36-59y** | 89 (8-274) | 102 (16-285) | 139 (27-307) | 234 (100-540) |
|  | **>=60y** | 67 (7-207) | 126 (19-315) | 156 (30-317) | 256 (99-580) |
| **Ischemic time, hours (median [IQR])** | **<1y** | 5.1 (4.3-5.8) | 5.5 (4.4-6.1) | 5.9 (5.3-6.6) | 5.9 (5.8-6.4) |
|  | **1-5y** | 5 (4.3-5.6) | 5.8 (4.8-6.6) | 6 (5.4-6.3) | 5.1 (4.1-6.1) |
|  | **6-10y** | 4.8 (3.3-5.6) | 5.3 (4.4-6) | 6 (5.1-6.8) | 5.8 (4.7-6.5) |
|  | **11-17y** | 5 (3.1-5.8) | 5.2 (4-6.1) | 5.5 (4.8-6.4) | 6.3 (5.4-7.3) |
|  | **18-35y** | 5.2 (3.9-6.5) | 5.4 (4.4-6.5) | 5.7 (4.7-6.8) | 6.8 (5.4-8.5) |
|  | **36-59y** | 4 (3.1-5.2) | 4.8 (3.8-6) | 5.4 (4.3-6.4) | 6.5 (5.3-8.4) |
|  | **>=60y** | 3.8 (3-4.8) | 4.5 (3.5-5.6) | 5 (4-6.1) | 6.2 (4.9-8.1) |
| **CPRA (mean ± SD)** | **<1y** | 0 ± NA | 0 ± NA | 12.4 ± 25.2 | 19 ± 24 |
|  | **1-5y** | - | - | 4.9 ± 15.5 | 34.2 ± 40.3 |
|  | **6-10y** | - | - | 19.3 ± 30 | 13.4 ± 19.4 |
|  | **11-17y** | - | - | 7 ± 13.8 | 12.1 ± 22 |
|  | **18-35y** | - | 0 ± NA | 8.7 ± 19.2 | 11.8 ± 22.5 |
|  | **36-59y** | - | 0 ± NA | 11.4 ± 23.8 | 14 ± 25.7 |
|  | **>=60y** | - | - | 8.9 ± 19.9 | 11.6 ± 23.3 |
| **Primary Graft Dysfunction (%)** | **<1y** | 1 (1.6%) | 3 (4.9%) | 6 (10.2%) | 0 (0%) |
|  | **1-5y** | 0 (0%) | 2 (7.1%) | 0 (0%) | 0 (0%) |
|  | **6-10y** | 2 (2.5%) | 5 (6%) | 6 (9.8%) | 0 (0%) |
|  | **11-17y** | 3 (1.2%) | 19 (5%) | 6 (2.2%) | 0 (0%) |
|  | **18-35y** | 14 (1.1%) | 88 (4.6%) | 38 (1.8%) | 16 (2.8%) |
|  | **36-59y** | 69 (1.6%) | 322 (4.6%) | 141 (1.9%) | 46 (1.3%) |
|  | **>=60y** | 30 (2.9%) | 205 (4.1%) | 190 (1.6%) | 65 (0.9%) |
| **Length of stay, days (median [IQR])** | **<1y** | 76 (62-79) | 43 (28-76) | 46 (30-87) | 62 (41-66) |
|  | **1-5y** | 2 (2-2) | 43 (18-82) | 19 (16-34) | 71 (48-92) |
|  | **6-10y** | 39 (39-39) | 22 (13-32) | 20 (12-40) | 22 (14-43) |
|  | **11-17y** | 29 (19-34) | 17 (12-24) | 18 (14-29) | 26 (16-35) |
|  | **18-35y** | 17 (14-26) | 17 (12-27) | 18 (13-29) | 22 (15-38) |
|  | **36-59y** | 15 (10-25) | 15 (10-25) | 18 (12-31) | 21 (14-36) |
|  | **>=60y** | 18 (11-33) | 15 (10-25) | 17 (11-28) | 19 (13-32) |
| **Graft failure (%)** | **<1y** | 50 (80.6%) | 42 (68.9%) | 31 (52.5%) | 4 (40%) |
|  | **1-5y** | 29 (87.9%) | 21 (75%) | 11 (55%) | 3 (50%) |
|  | **6-10y** | 68 (86.1%) | 64 (77.1%) | 27 (44.3%) | 4 (23.5%) |
|  | **11-17y** | 227 (88%) | 321 (84.7%) | 176 (64.2%) | 12 (18.8%) |
|  | **18-35y** | 1130 (87.5%) | 1506 (79.3%) | 1185 (55.5%) | 113 (20.1%) |
|  | **36-59y** | 3997 (93.5%) | 5974 (85.3%) | 4020 (52.8%) | 543 (15%) |
|  | **>=60y** | 991 (95.5%) | 4672 (93.5%) | 7359 (62.8%) | 1202 (16.8%) |
| **Chronic Rejection as Death Cause (%)** | **<1y** | 5 (8.1%) | 13 (21.3%) | 10 (16.9%) | 1 (10%) |
|  | **1-5y** | 6 (18.2%) | 6 (21.4%) | 7 (35%) | 0 (0%) |
|  | **6-10y** | 11 (13.9%) | 32 (38.6%) | 9 (14.8%) | 0 (0%) |
|  | **11-17y** | 14 (5.4%) | 132 (34.8%) | 66 (24.1%) | 4 (6.2%) |
|  | **18-35y** | 85 (6.6%) | 426 (22.4%) | 385 (18%) | 22 (3.9%) |
|  | **36-59y** | 235 (5.5%) | 1163 (16.6%) | 897 (11.8%) | 56 (1.5%) |
|  | **>=60y** | 42 (4%) | 709 (14.2%) | 1045 (8.9%) | 81 (1.1%) |
| **Post-TX Dialysis (%)** | **<1y** | 6 (9.7%) | 3 (4.9%) | 2 (3.4%) | 0 (0%) |
|  | **1-5y** | 2 (6.1%) | 0 (0%) | 0 (0%) | 0 (0%) |
|  | **6-10y** | 4 (5.1%) | 5 (6%) | 1 (1.6%) | 0 (0%) |
|  | **11-17y** | 25 (9.7%) | 22 (5.8%) | 15 (5.5%) | 3 (4.7%) |
|  | **18-35y** | 175 (13.5%) | 180 (9.5%) | 136 (6.4%) | 9 (1.6%) |
|  | **36-59y** | 529 (12.4%) | 743 (10.6%) | 643 (8.4%) | 110 (3%) |
|  | **>=60y** | 108 (10.4%) | 491 (9.8%) | 880 (7.5%) | 177 (2.5%) |
| **Post-TX Diabetes (%)** | **<1y** | 0 (0%) | 0 (0%) | 1 (1.7%) | 0 (0%) |
|  | **1-5y** | 1 (3%) | 2 (7.1%) | 0 (0%) | 0 (0%) |
|  | **6-10y** | 16 (20.3%) | 23 (27.7%) | 7 (11.5%) | 0 (0%) |
|  | **11-17y** | 102 (39.5%) | 173 (45.6%) | 32 (11.7%) | 8 (12.5%) |
|  | **18-35y** | 510 (39.5%) | 816 (42.9%) | 301 (14.1%) | 31 (5.5%) |
|  | **36-59y** | 1015 (23.7%) | 2382 (34%) | 1095 (14.4%) | 175 (4.8%) |
|  | **>=60y** | 217 (20.9%) | 1627 (32.6%) | 1639 (14%) | 376 (5.3%) |
| **Post-TX Malignancy (%)** | **<1y** | 7 (11.3%) | 8 (13.1%) | 1 (1.7%) | 0 (0%) |
|  | **1-5y** | 2 (6.1%) | 4 (14.3%) | 0 (0%) | 1 (16.7%) |
|  | **6-10y** | 7 (8.9%) | 8 (9.6%) | 6 (9.8%) | 0 (0%) |
|  | **11-17y** | 26 (10.1%) | 41 (10.8%) | 22 (8%) | 2 (3.1%) |
|  | **18-35y** | 195 (15.1%) | 313 (16.5%) | 181 (8.5%) | 11 (2%) |
|  | **36-59y** | 762 (17.8%) | 2028 (28.9%) | 1612 (21.2%) | 102 (2.8%) |
|  | **>=60y** | 203 (19.6%) | 1755 (35.1%) | 3528 (30.1%) | 378 (5.3%) |
